# Identification of *Candida* species isolated from vulvovaginitis using matrix assisted laser desorption ionization-time of flight mass spectrometry

**DOI:** 10.29252/cmm.3.4.21

**Published:** 2017-12

**Authors:** Majid Alizadeh, Anna Kolecka, Teun Boekhout, Hossein Zarrinfar, Mohamad. A Ghanbari Nahzag, Parisa Badiee, Ali Rezaei-Matehkolaei, Abdolmajid Fata, Somayeh Dolatabadi, Mohammad. J Najafzadeh

**Affiliations:** 1 Department of Parasitology and Mycology, Faculty of Medicine, Mashhad University of Medical Sciences, Mashhad, Iran; 2 Westerdijk Fungal Biodiversity Institute, Utrecht, The Netherlands; 3 Institute for Biodiversity and Ecosystem Dynamics (IBED), University of Amsterdam, Amsterdam, The Netherlands; 4 Allergy Research Center, Mashhad University of Medical Sciences, Mashhad, Iran; 5 Alborzi Clinical Microbiology Research Center, Shiraz University of Medical Sciences, Shiraz, Iran; 6 Department of Medical Mycology, School of Medicine, Health Research Institute, Infectious and Tropical Diseases Research Center, Ahvaz Jundishapur University of Medical Sciences, Ahvaz, Iran; 7 Department of Parasitology and Mycology, Research Center for Cutaneous Leishmaniasis, School of Medicine, Mashhad University of Medical Sciences, Mashhad, Iran; 8 Faculty of Engineering, Sabzevar University of New Technologies, Sabzevar, Iran; 9 Cancer Molecular Pathology Research Center, Mashhad University of Medical Sciences, Mashhad, Iran

**Keywords:** Candidiasis, Identification, MALDI-TOF MS, Vulvovaginitis

## Abstract

**Background and Purpose:**

Vulvovaginal candidiasis (VVC) is a common problem in women. The purpose of this study was to identify *Candida* isolates by matrix assisted laser desorption ionization-time of flight mass spectrometry (MALDI-TOF MS) from women with vulvovaginitis that were referred to Ghaem Hospital, Mashhad, Iran.

**Materials and Methods:**

This study was conducted on 65 clinical samples isolated from women that were referred to Ghaem Hospital. All specimens were identified using phenotyping techniques, such as microscopy and culture on Sabouraud dextrose agar and corn meal agar. In addition, all isolates were processed for MALDI-TOF MS identification.

**Results:**

Out of the 65 analyzed isolates, 61 (94%) samples were recognized by MALDI-TOF MS. However, the remaining four isolates (6%) had no reliable identification. According to the results, *C. albicans* (58.5%) was the most frequently isolated species, followed by *C. tropicalis* (16.9%), *C. glabrata* (7.7%), *C. parapsilosis* (7.7%), and *guilliermondii *(3.1%).

**Conclusion:**

As the findings indicated, MALDI TOF MS was successful in the identification of clinical *Candida* species. *C. albicans* was identified as the most common *Candida* species isolated from the women with VVC. Moreover, *C. tropicalis* was the most common species among the non-*albicans Candida* species.

## Introduction

Genus *Candida* includes more than 300 species and belongs to the kingdom fungi, Phylum Ascomycota, Subphylum Saccharomycotina, class Saccharomycetes, and order Saccharomycetales [1]. Within the last two decades, the incidence of infections due to *Candida* species has significantly increased [2-4]. *Candida* is carried by nearly half of the population as a harmless commensal [5]. In healthy individuals, the *Candida* colonization of body sites, including oral cavity, skin, gastrointestinal tract, and vaginal surfaces is normal [6, 7]. 

Vulvovaginal candidiasis (VVC) is due to the overgrowth of *Candida* yeast species within the vagina, which is characterized by curd-like vaginal discharge, erythema, and itching [8]. The VVC is a vaginal infection, affecting approximately 75% of the females of child-bearing age one or more times in their lifetime. This infection can occur recurrently in approximately 5% of the females [9, 10]. 

The VVC can not only affect the woman, but also girls. The increased susceptibility of children to VVC could be explained by several factors. These factors include anatomical features of the children (i.e., proximity to the rectum, pubic hair, small labia minora, lack of labial fat pads, thin and delicate vulvar skin, as well as thin, atrophic, and anoestrogenic vaginal mucosa), children’s tendency to explore their bodies, and poor local hygiene [11, 12]. *C. albicans* accounts for 80-92% of all cases of candidiasis infections worldwide, especially VVC [13-15]. However, the recurrence of non-*albicans* *Candida* species, especially *C. glabrata*, *C. parapsilosis,* and *C. tropicalis* has increased in the healthy females [16].

Most of the commonly isolated yeast species can be identified by the commercially available biochemical test systems. However, the rarely occurring species often remains unidentified or misidentified [17-19]. Additionally, phenotypic tests require 1-3 days of incubation to obtain results. To overcome the inaccuracies of biochemical identification methods, molecular methods have been developed. 

The molecular identification tests for yeasts are based on a polymerase chain reaction amplification, followed by the sequencing of the internal transcribed spacer region and the D1/D2 domains of the large subunit of the ribosomal DNA [20-23]. While these assays are highly accurate, they require considerable processing time and expensive reagents.

As an alternative to biochemical and molecular identification schemes, matrix assisted laser desorption ionization time-of-flight mass spectrometry (MALDI-TOF MS) can be used for the reliable identification of *Candida* species. Moreover, this method has been recently used for the routine identification of microorganisms, especially bacteria and yeasts, in clinical centers in several parts of the world [24-26]. The MALDI-TOF MS facilitates the identification of a large spectrum of proteins directly from intact microorganisms [27]. 

This method has been shown to precisely detect bacteria and yeast at the species level [28]. Within this technique, spectra are compared to a reference database, and recognition is acquired by matching the unknown spectrum to the most similar spectrum in the database to identify the unknown microorganism [29]. With this background in mind, the present study was performed to identify *Candida* species from women suffering from vulvovaginitis that were referred to Ghaem Hospital, Mashhad, Iran, by means of MALDI-TOF MS.

## Materials and Methods

This study was conducted on 65 samples obtained from the women with VVC through routine measures at Ghaem Hospital Mashhad, Iran. The exclusion criteria were negative culture result and contamination by saprobic fungi. This study was approved by the Ethics Committee of Mashhad University of Medical Sciences, Mashhad, Iran (ethical code: IR.MUMS.REC.1393.762).

***Purification of Candida colonies***

All isolates were cultured onto Sabouraud dextrose agar and incubated for 2-3 days at 30°C. The isolates were stained by Giemsa staining. A germ tube test and the formation of chlamydospores were assessed for the confirmation of the genus *Candida* [30].

***Matrix assisted laser desorption ionization time-of-flight mass spectrometry identification***

All yeast isolates were subcultured on Sabouraud dextrose agar plates and incubated at 30°C for 24 h. Yeast proteins were extracted according to the Bruker Daltonics GmbH protocol using the formic acid/ethanol extraction protocol [31, 32]. Briefly, a single colony of the sample isolates were suspended in 300 µL of Milli-Q water. A formic acid volume of 30-50 µL was found to be optimal, and an equal volume of acetonitrile was added later on. 

From the crude protein extracted from each tested strain, 1 µL was spotted in duplicate on a 96-spot polished steel target plate (Bruker Daltonics, Bremen, Germany) and allowed to air drying. The bacterial test standard was utilized as a positive control (Bruker Daltonics). Prior to analysis, all tested spots were overlaid with 1 μL of alpha-cyano-4-hydroxycinnamic acid (Bruker Daltonics). After drying at room temperature they were analyzed in automatic runs operated by flexControl, version 3.3.108.0 (Bruker Daltonics). 

The yeast identification was accomplished by means of MALDI-TOF MS Biotyper RTC software, version 3.0 (Bruker Daltonics), based on the comparison of mass spectra generated by the Microflex LT software with databases. The recognition results were scored according to the manufacturer’s criteria. In this regard, the log score values of > 2.0, 1.7-2, and < 1.7 indicated correct species identification, correct genus identification, and no reliable identification, respectively. The obtained mass spectra were visualized as described by Cendejas-Bueno et al. [33]. The identification was considered precise if at least one spot from the duplicates had a score of > 1.7 and gave reliable identification.

## Results

*Candida* isolates obtained from VVC could not be entirely recognized at the species level by biochemical methods. Out of the 65 analyzed isolates, 93.8% of the samples were identified by MALDI-TOF MS (Table 1). According to the results, *C. albicans* was the most frequently isolated species (58.5 %, n=38), while other *Candida* species comprised 41.5% (n=27) of the samples. These species included *C. glabrata* (7.7 %, n=5), *C. tropicalis* (16.9%, n=11), *C. parapsilosis* (7.7%, n=5), and *Meyerozyma guilliermondii* (3.1%, n=2). The MALDI-TOF MS failed to correctly identify four species (6.1 %, n=4). 

Overall, 58 (89.2%), 3 (4.6%), and 4 (6.2%) isolates had correct species identification level (i.e., log score value of >2.0), genus level (log score value of 1.7-2.0), and no reliable identification (log score value of <1.7) respectively. The performance of MALDI -TOF MS for the routine identification of the clinical isolates is tabulated in Table 1.

**Table 1:** Results of matrix assisted laser desorption ionization-time of flight mass spectrometry identification of 65 isolates analyzed with Bruker Daltonics

**Candida spp.**	**No. of analyzed isolates (%)**	**No. with MALDI-TOF score**		
		**>2.0**	**1.7-2.0**	**<1.7**
*Candida albicans*	38 (58.5)	37	1	
*Candida tropicalis*	11 (16.9)	9	2	
*Candida glabrata*	5 (7.7)	5		
*Candida parapsilosis*	5 (7.7)	5		
*Meyerozyma guillermondii*	2 (3.1)	2		
Species not included in database	4 (6.1)			4
Total	65	58	3	4

MALDI-TOF: matrix assisted laser desorption ionization-time of flight mass spectrometry

## Discussion

In the present study, we used MALDI-TOF MS for the identification of *Candida* species isolated from VVC and evaluated the prevalence of each species. Although the conventional biochemical screening for yeast recognition is cheap, it is time-consuming and occasionally incorrect [17-19]. On the other hand, gene sequencing is precise, but high-priced, time-consuming, technically demanding, and not always available for routine identification [23]. 

Bacteria cause 40-50% of cases of vaginal infections, and *Candida* species accounts for 20-25% of the cases [14]. The pathogenesis of VVC due to *Candida* species is complex and depends on host factors and fungal species involved. These factors may increase the recurrent experience of candidiasis and make the treatment process more complicated [34, 35]. However, the correct identification of *Candida* species and targeted antifungal therapy can reduce antifungal drug resistance in these species. Among the clinically relevant *Candida* species, *C. albicans* has a low azole resistance rate. Nonetheless, the prevalence of other species, such as *C. krusei* and *C. glabrata*, which are resistant to triazole and especially fluconazole is on a rising trend [36].

The MALDI-TOF MS as a new technique has emerged for the identification of clinical yeast and yeast-like isolates. The utilization of either on-plate extraction or standard tube-based methods provides a precise identification for most of the yeast isolates within 10-40 min with only 5 min of hands-on time [32, 37]. Preparation for MALDI-TOF MS analysis, including protein extraction, requires almost cheap reagents [37]. While the MALDI-TOF MS instrument is expensive, the use of cheap reagents and high recognition rates significantly decrease the cost for the identification of organisms in comparison to the current techniques [37].

In addition to the detection of *Candida* species, this technique has also been used for the identification of other fungal species, such as other yeasts [26, 27, 38-41], dermatophyte species [42-44], and filamentous fungi, like *Aspergillus *species [27, 45-47]. In line with the literature, in this study *C. albicans* (58.5%) was the most prevalent species associated with VVC [15]. Likewise, in an epidemiologic study of VVC cases, *C. albicans *was the most prevalent cause as well [48].

The incidence of *C. albicans *in pregnant woman is higher than in non-pregnant ones. This is due to the fact that *C. albicans* is able to adhere to vaginal epithelium more readily than other *Candida* species [49]. In our study, 94% of the isolates were recognized by MALDI-TOF MS. Similarly, in another study on clinical yeast isolates, MALDI-TOF MS facilitated the identification of 92.5% of samples [32]. Pulcrano et al. [50] and Yaman et al. [51] could identify 100% of *Candida* isolates through this method. Based on the findings, it can be concluded that MALDI-TOF MS is a rapid and accurate technique for the identification of *Candida* species isolated from VVC.

## Conclusion

As the findings of the present study indicated, MALDI TOF MS method was successfully used for the identification of clinical *Candida* species. In the current study, *C. albicans* and* C. tropicalis* were identified as the dominant yeast* Candida* species isolated from women with VVC, respectively.
